# 靶向B细胞和浆细胞的CAR-T细胞治疗中防治乙型肝炎病毒再激活的中国专家共识（2021年版）

**DOI:** 10.3760/cma.j.issn.0253-2727.2021.06.001

**Published:** 2021-06

**Authors:** 中国抗癌协会血液肿瘤专业委员会 中华医学会血液学分会

近年来，嵌合抗原受体T细胞（CAR-T）疗法在治疗难治/复发B细胞非霍奇金淋巴瘤（B-NHL）、急性B淋巴细胞白血病（B-ALL）、多发性骨髓瘤（MM）等多种血液肿瘤中取得了突破性进展[1]–[4]。其中，针对B-ALL、大B细胞淋巴瘤、套细胞淋巴瘤、滤泡淋巴瘤和MM的CAR-T细胞产品已在多个国家正式进入临床应用。在我国，针对CD19、CD22、CD20、BCMA、CD38、CD30等多个靶点的CAR-T细胞疗法也在多家临床中心开展多项临床试验[3],[5]–[8]。已有多项研究报道靶向B细胞和浆细胞的CAR-T细胞治疗可诱发HBV再激活，少数病例甚至引起暴发性肝炎、肝衰竭，甚至死亡[9]–[17]。在我国，合并乙型肝炎病毒（HBV）感染的血液肿瘤患者人数众多，为在这些患者中安全地开展CAR-T细胞治疗，中国抗癌协会血液肿瘤专业委员会和中华医学会血液学分会组织制定了本专家共识。此共识在防治血液肿瘤患者HBV再激活相关指南和专家共识的基础上[18]–[25]，纳入我国多家临床中心在CAR-T细胞治疗后HBV再激活方面的主要研究成果和临床经验[9]–[13],[15]–[16]，目的是在此新领域迅速形成符合我国情况的诊疗规范，为各中心开展CAR-T细胞治疗提供指导性意见。

一、相关概念[18]–[27]

1. 慢性HBV感染：乙型肝炎表面抗原（HBsAg）阳性的患者。

2. 慢性乙型肝炎：由HBV持续感染引起的肝脏慢性炎症性疾病。

3. 乙型肝炎康复：HBsAg阴性、HBV核心抗体（即抗-HBc）阳性、HBV表面抗原抗体（即抗-HBs）阳性或阴性、丙氨酸转氨酶（ALT）在正常范围。

4. HBV再激活：在接受免疫抑制治疗或化学治疗时，慢性HBV感染者或乙型肝炎康复者HBV DNA较基线升高≥100倍，或基线HBV DNA阴性者转为阳性，或HBsAg由阴性转为阳性。

5. 病毒学突破：核苷（酸）类似物（NAs）治疗依从性良好的患者，在未更改治疗的情况下，HBV DNA水平比治疗中最低值升高>10倍，或HBV DNA阴性又转为阳性，可有或无ALT升高。

6. 耐药：在抗病毒治疗过程中，检测到与HBV耐药相关的基因突变，称为基因型耐药。体外实验显示，抗病毒药物敏感性降低，并与基因耐药相关，称为表型耐药；针对1种抗病毒药物出现的耐药突变对另外l种或几种抗病毒药物也出现耐药，称为交叉耐药；至少对2种不同类别的NAs耐药，称为多重耐药。

7. B淋巴细胞消减：外周血B淋巴细胞低于白细胞总数的1％，或低于淋巴细胞总数的3％，即低于外周血B淋巴细胞的正常低限。

8. B淋巴细胞恢复：外周血B淋巴细胞≥白细胞总数的1％，或≥淋巴细胞总数的3％，即超过外周血B淋巴细胞的正常低限。

二、淋巴瘤治疗中的HBV再激活

研究表明慢性HBV感染会显著增加包括弥漫大B细胞淋巴瘤（DLBCL）在内的NHL患病风险[28]，且合并HBV感染的DLBCL患者有独特的临床和组学特征[29]，提示HBV感染能促进DLBCL等NHL的发生。在我国，9％～30％的NHL（包括25％的DLBCL）患者为HBV慢性感染者[20],[30]–[32]，20％～44％的DLBCL患者为乙型肝炎康复者[20],[33]–[34]。HBV感染在NHL，尤其是DLBCL患者中的发生率明显高于其他肿瘤患者，相应的，NHL患者在治疗后会有更高的风险发生HBV再激活[35]–[36]。

HBV再激活的风险随患者HBV血清学状况和治疗方式的不同而异[23]–[24]。HBsAg阳性和HBV DNA阳性是HBV再激活的病毒学危险因素。接受造血干细胞移植或化学免疫治疗（包括利妥昔单抗和糖皮质激素的化疗方案）的HBV慢性感染者和乙型肝炎康复者是发生HBV再激活的高危人群[23]–[24],[36]–[38]。由于利妥昔单抗可诱导持续的B细胞消减和体液免疫缺陷[39]–[40]，在接受化学免疫治疗的B-NHL患者中，21％～60％的HBV慢性感染者和2％～41.5％的乙型肝炎康复者会发生HBV再激活[20],[36],[41]。随机对照临床研究表明，在这些接受化学免疫治疗的血液肿瘤患者中，预防性使用NAs能将HBV慢性感染者和乙型肝炎康复者病毒再激活的风险降至2.1％～6.6％[24],[36],[42]–[44]。

三、CAR-T细胞治疗相关的HBV再激活CAR-T细胞治疗可以诱导持久的B淋巴细胞消减。在CTL019治疗有效的DLBCL患者中，B淋巴细胞恢复的中位时间为回输后6.7个月[26]–[27]。因此，CAR-T细胞治疗同样是诱发HBV再激活的高危因素。国内外多个中心报道了HBV慢性感染和乙型肝炎康复者在接受抗CD19、CD22或BCMA CAR-T细胞治疗后发生HBV再激活的病例[9]–[10],[13],[15]–[17]。这些患者可表现为无症状性HBV DNA拷贝数升高或HBsAg血清学转换，严重者出现肝炎症状，甚至因暴发性肝衰竭而死亡[9]–[10],[13],[15]–[17]。HBV再激活多发生在CAR-T细胞输注后6个月内，但也有在CAR-T输注后16个月发生再激活的报道[9]–[10],[13],[15]–[17]。对HBV慢性感染者，即便在CAR-T细胞治疗中预防性使用包括恩替卡韦（ETV）在内的NAs，仍有5.3％～20％的病例发生HBV再激活。大多仅表现为无症状的HBV DNA拷贝数升高[9]–[10]，肝炎的风险约为5.3％[15]。对乙型肝炎康复者，HBV再激活发生在3.4％～16.7％未接受NAs预防的乙型肝炎康复者中，但能被NAs及时控制[10]。预防性使用NAs可能会降低CAR-T细胞治疗相关的HBV再激活风险。但过早停用NAs能激活HBV，严重者造成暴发性肝炎甚至肝衰竭[9],[16]–[17]。及时发现并重启NAs治疗，仍能有效控制病毒再激活[9],[17]。

四、合并HBV感染对CAR-T细胞治疗的影响

动物试验和大多数临床研究表明，HBV感染并没有影响CAR-T细胞的活性和临床疗效；没有明显升高CAR-T细胞治疗中血清转氨酶、胆红素、IL-6和C-反应蛋白的水平；也没有增加细胞因子释放综合征（CRS）和免疫效应细胞相关神经毒性综合征的发生率或严重程度[10],[12],[15]。因此，目前并没有确切的证据提示HBV感染会影响CAR-T细胞疗法的安全性和有效性。

五、CAR-T细胞治疗相关HBV再激活的防治策略

1. 预防HBV再激活的治疗前筛查：应仔细询问拟行CAR-T细胞治疗的血液肿瘤患者既往肝脏疾病史和用药治疗史。治疗前应常规筛查HBV血清学标志物（HBsAg、HBsAb和HBcAb）、HBV DNA和肝功能（ALT、天冬氨酸转氨酶、胆红素、白蛋白、凝血酶原时间等）。对于既往抗肿瘤治疗期间已预防性应用NAs的患者，仔细询问其既往NAs使用情况及抗肿瘤期间肝功能和HBV-DNA状况。

2. CAR-T相关HBV再激活的预防策略：HBV慢性感染者和乙型肝炎康复者是发生CAR-T细胞治疗相关HBV再激活的高危人群。对于HBV慢性感染者和HBV DNA阳性的乙型肝炎康复者，应预防性使用NAs（图1）[10],[15]。对于HBV DNA阴性的乙型肝炎康复者，可预防性使用NAs；若未给予NAs预防，应严密监测HBV再激活，及时发现并治疗可能发生的病毒激活（图1）[20]–[24]。对于HBV监测依从性差的乙型肝炎康复者，建议进行抗病毒预防[10],[13]。有研究指出HBsAb<56.48 IU/L且HBcAb≥6.41×10^3^ IU/L的乙型肝炎康复者易发生HBV再激活[45]–[47]，对此类具有危险因素的乙型肝炎康复者同样可以预防性使用NAs。此外，慢性乙型肝炎患者应谨慎评估其接受CAR-T细胞治疗的风险，其NAs抗病毒治疗的疗程、随访监测和停药原则应参考中华医学会感染病学分会和中华医学会肝病学分会制定的《慢性乙型肝炎防治指南（2019年版）》对于普通慢性乙型肝炎患者的诊疗建议[21]。

**图1:**
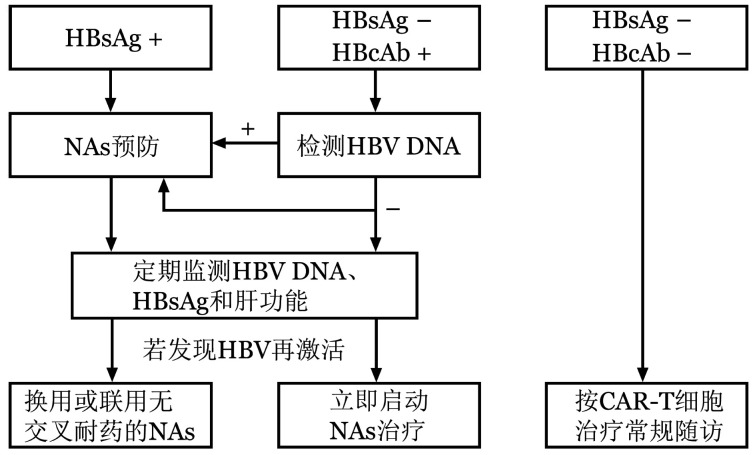
CAR-T相关HBV再激活的预防策略 CAR-T：嵌合抗原受体T细胞；NAs：核苷（酸）类似物

3. 预防性使用NAs的药物选择和疗程：预防性NAs应在预处理前1周给予（图2），应首选强效且低耐药的NAs，包括ETV、富马酸替诺福韦酯（TDF）或富马酸丙酚替诺福韦（TAF）。不建议使用拉米夫定（LAM）或阿德福韦酯（ADV）。对于曾经使用过LAM或替比夫定（LdT）的HBV感染者，因易出现对ETV的耐药，应选择无交叉耐药的TDF或TAF预防（无交叉耐药NAs的选择可参考文献[21]）[20]–[25]。

**图2:**
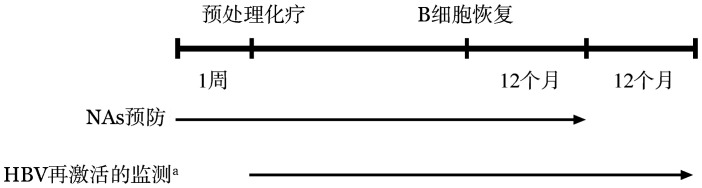
预防性使用NAs和监测HBV再激活的时程 NAs：核苷（酸）类似物。^a^若未预防性使用NAs，HBV再激活的监测应持续至外周血B淋巴细胞恢复后12个月

由于不同个体接受CAR-T细胞输注后，B淋巴细胞消减持续的时间并不一致[26]–[27]，预防性使用NAs的疗程应取决于患者B淋巴细胞消减持续的时间。我们建议监测患者外周血淋巴细胞亚群，当出现外周血B淋巴细胞恢复后继续预防性使用NAs 12个月（图2）[20]–[25]。

4. HBV再激活的监测与停药：对于接受NAs预防的HBV慢性感染者和乙型肝炎康复者，应每1～3个月监测HBV DNA、HBV血清学标志物和肝功能，以便及时发现并应对HBV再激活。HBV DNA定量采用实时定量PCR。HBV再激活多发生在CAR-T细胞输注后6个月内[9]–[10],[13],[15]，在接受NAs预防期间，应每月监测1次上述指标，半年后每3个月监测1次（图2）[20]–[24]。因有停用NAs半年内发生HBV再激活的案例[9],[16]–[17]，故在停用NAs预防后仍应继续监测上述指标，停用NAs半年内每1个月监测1次，半年后每3个月监测1次，直至停用NAs后12个月（图2）[9],[16]–[17]。

对于未接受NAs预防的乙型肝炎康复者，更应积极采用上述监测方法，直至外周血B淋巴细胞恢复后12个月[20]–[24]。

5. HBV再激活的治疗：若发生HBV再激活，应在复核检查结果的同时，立即启动或调整NAs治疗。对未预防性使用NAs的患者，无论ALT是否升高，均应立即启动NAs治疗（图1），尽可能将HBV再激活控制在HBV DNA拷贝数上升的早期无症状阶段，以避免发展为HBV相关肝炎，甚至因急性肝衰竭而危及生命，即抢先治疗。对已使用NAs预防的患者，应立即换用或联用无交叉耐药的NAs治疗（图1），即挽救治疗。采用ETV者换用TDF或TAF，采用TDF或TAF者换用ETV，或2种药物联合使用（无交叉耐药NAs的选择可参考文献[21]）[20]–[25]。

此外，发生病毒性突破的患者，除上述治疗外，还可进行HBV耐药突变检测，尤其是既往对拉米夫定耐药的患者，他们更易被诱导出对ETV交叉耐药的突变。建议咨询肝病科医师指导抗病毒治疗方案。发生HBV相关肝炎或肝衰竭的患者，应与淋巴瘤、其他病原、药物或毒物、酒精、脂肪肝、自身免疫和代谢性疾病等病因相鉴别，并与肝病科积极协作进行治疗。

六、其他需要关注的问题

我们需要评估患者年龄、系统性疾病和其他肝脏问题及其治疗对HBV再激活的影响。对复杂病例，应进行影像学、内窥镜和肝纤维化的检查，以全面评估包括肝硬化、门静脉高压在内的治疗风险，并及时咨询肝病科医师。应结合病史、实验室和影像学检查结果及患者依从性，综合考虑患者HBV再激活的危险因素，采用合适的防治策略。

我们需要做好患者教育，提高患者接受NAs预防和HBV再激活监测的依从性，帮助医师和患者及时识别NAs少见的不良反应（肾功能不全、低磷性骨病、肌炎、横纹肌溶解、乳酸酸中毒等），避免不规范治疗和长期用药导致的风险[20]–[21]。此外，我国已批准TAF（≥12岁且体重≥35 kg）用于治疗青少年慢性乙型肝炎，美国已批准ETV（≥2岁）和TDF（≥2岁且体重≥10 kg）用于治疗儿童慢性乙型肝炎[21]。由于儿童血液肿瘤患者CAR-T细胞治疗中的HBV再激活目前还缺乏报道，防治建议咨询儿科和肝病科医师，并参考相关指南和药物说明书调整剂量。

由于CAR-T细胞治疗是细胞免疫治疗新技术，国内外各中心积累的病例和经验相对有限，我们需要谨慎地解读此专家共识，并在临床实践中不断加以检验和更新，以提高我们对相关临床问题的认识和处理水平。
